# BodyFlow: An Open-Source Library for Multimodal Human Activity Recognition

**DOI:** 10.3390/s24206729

**Published:** 2024-10-19

**Authors:** Rafael del-Hoyo-Alonso, Ana Caren Hernández-Ruiz, Carlos Marañes-Nueno, Irene López-Bosque, Rocío Aznar-Gimeno, Pilar Salvo-Ibañez, Pablo Pérez-Lázaro, David Abadía-Gallego, María de la Vega Rodrigálvarez-Chamarro

**Affiliations:** Department of Big Data and Cognitive Systems, Instituto Tecnológico de Aragón (ITA), María de Luna 7-8, 50018 Zaragoza, Spain; rdelhoyo@ita.es (R.d.-H.-A.); ahernandez@ita.es (A.C.H.-R.); cmaranes@ita.es (C.M.-N.); ilopez@ita.es (I.L.-B.); psalvo@ita.es (P.S.-I.); plazaro@ita.es (P.P.-L.); dabadia@ita.es (D.A.-G.)

**Keywords:** multimodal human activity recognition, human pose estimation, deep learning, sensors

## Abstract

Human activity recognition is a critical task for various applications across healthcare, sports, security, gaming, and other fields. This paper presents BodyFlow, a comprehensive library that seamlessly integrates human pose estimation and multiple-person estimation and tracking, along with activity recognition modules. BodyFlow enables users to effortlessly identify common activities and 2D/3D body joints from input sources such as videos, image sets, or webcams. Additionally, the library can simultaneously process inertial sensor data, offering users the flexibility to choose their preferred input, thus facilitating multimodal human activity recognition. BodyFlow incorporates state-of-the-art algorithms for 2D and 3D pose estimation and three distinct models for human activity recognition.

## 1. Introduction

Human activity recognition (HAR) is the field of study focused on identifying activities of daily human life, such as walking, sitting, or falling, using artificial intelligence (AI) on data collected through various sources or devices. Examples of such devices include wearable sensors [1] that integrate low-power sensors to detect motion or other physiological signals, inertial sensors, and video cameras [2]. The use of multiple information sources makes HAR useful in applications such as healthcare, surveillance, smart homes, or movement monitoring for athletes.

In healthcare, HAR can bring significant benefits by assisting elderly individuals who are facing autonomy challenges and desire to maintain a normal lifestyle at their own homes but need continuous real-time support [3,4,5,6]. Recent surveys highlight the potential of wearable sensor modalities in supporting older adults’ daily activities [7]. Additionally, HAR has great potential for the early detection of health issues and improving quality of life [8]. For example, a HAR system could identify falls and provide real-time alerts, enabling prompt intervention to prevent serious complications [1,9,10]. Furthermore, HAR can be used not only for immediate detection but also for the continuous monitoring of behavior, allowing for the identification of significant changes in the user’s behavior. For instance, it is possible to track a patient’s therapy routine after an accident or monitor their condition at home. HAR systems could detect events that, although common, may reflect unusual behaviors depending on the user and the temporal context [5]. For example, if a user who normally walks is detected as ‘sitting’ or ‘standing’ without moving for an extended period, this could indicate a health problem, such as a stroke or disorientation. Moreover, physical inactivity, which is a significant risk factor for chronic diseases, can also be identified through HAR algorithms [11]. This technology enables the monitoring of walking activity and can detect periods of movement, revealing differences, deterioration, or anomalies associated with neurodegenerative diseases such as Parkinson’s [12], early dementia [13], or sarcopenia [14] through gait data analysis. Beyond healthcare, HAR also plays a critical role in workplace safety and accident prevention. Accident recognition in the workplace is another common application for HAR [6]. With the rise of Industry 5.0, the focus on workplace well-being has expanded to not only prevent physical accidents but also monitor cognitive aspects of workers’ safety [15]. For example, it is useful to recognize a worker moving erratically or in a danger zone so that a potential accident or injury is avoided. Furthermore, recent innovations in smart workplace environments are adopting collaborative multimodal federated learning approaches, which enable HAR systems to leverage data from multiple devices while ensuring user privacy through decentralized model training [16].

The challenges of utilizing information in HAR tasks vary depending on the data source, be it sensors or cameras. Regarding vision-based human activity recognition, there are mainly two approaches: the one that directly uses RGB images or videos to identify the activity and the one that estimates the pose of the person [17]. This second approach, human pose estimation (HPE), is one of the most popular tasks in the field of computer vision where, together with artificial intelligence techniques, machines are able to detect and track body joints that describe the pose of a person.

In this work, BodyFlow is presented as a plug-and-play library designed to simplify the training of human activity recognition (HAR) models using visual data processed by human pose estimation algorithms and inertial data. The objective of the proposed work is to fulfill the demand for an open-source library that encompasses both human pose estimation (HPE), Multi-Person Pose Estimation, and multimodal HAR. The framework incorporates 2D and 3D state-of-the-art algorithms for HPE, providing flexibility through their combination. The user may only use the HPE module to estimate 2D/3D poses. We also allow Multi-Person Pose Estimation, enabling the detection and tracking of multiple individuals in various environments. This feature is crucial for applications in crowded or dynamic settings where understanding interactions between multiple people is necessary. Multi-Person Pose Estimation enhances the framework’s capability to handle complex scenarios. To further enhance customization, we allow users to choose between three different tracking algorithms, providing tailored solutions based on specific application needs. Additionally, BodyFlow enables HAR through three pretrained models, establishing a comprehensive solution for multimodal human activity recognition. The primary objective of the library is to simplify the utilization of intricate networks or algorithms, allowing for the straightforward training of HPE and HAR models. Additionally, HPE and HAR modules are designed to function collaboratively or as autonomous entities, according to the user’s needs.

This paper is organized into several sections. Firstly, a review of the current state of the art is presented. Then, a detailed description of the framework, including its modules and functions, follows. The results obtained by the framework with the UP-FALL dataset [18] are presented in Section 4. Finally, this paper concludes with a summary of the findings and suggestions for future research.

## 2. Related Work

### 2.1. Multimodal HAR

HAR techniques make use of different devices to collect information on human activities. These techniques can predict human activities using input data from wearable inertial sensors, video cameras, or a combination of both, a method commonly known as multimodal HAR [19].

Sensor-based human activity recognition involves using sensors such as accelerometers, gyroscopes, and magnetometers to capture data about a person’s movements and postures. The proposed work focuses on wearable sensors. These can be classified into physical sensors and physiological sensors [20]. Physical sensors include inertial measurement units (IMUs), piezoelectric sensors, GPS, wearable cameras, etc. On the other hand, physiological sensors monitor the responses of the human body, such as electromyography sensors.

In a typical sensor-based process for HAR [21,22], the initial step involves pre-processing raw temporal data obtained from sensors and subsequently segmenting the information [23]. Then, relevant features are extracted from those segments, which are used to train and evaluate an activity classifier, such as time-domain features (e.g., the mean and standard deviation), frequency-domain features (e.g., FFT components and spectral energy), and wavelet-based features (e.g., detail coefficients) [24]. Since the efficacy of the classification is directly impacted by the quality of the feature representation, the feature extraction stage has received the majority of recent attention, particularly in the context of sensor-based activity recognition, where deep learning techniques have been employed to automate high-level feature extraction, improving classification performance significantly [25]. Traditionally, features were handcrafted by specialists who had an understanding of the application domain; however, this approach is costly and not always possible in practice, which has driven the field of automatic feature learning [26]. Notably, Artificial Neural Networks (ANNs) have emerged as a prominent choice in this context, particularly due to their ability to automate feature extraction from raw sensor inputs while modeling temporal dependencies, as demonstrated by Ordóñez and Roggen’s work on combining deep convolutional and LSTM recurrent networks for human activity recognition [27]. ANNs are machine learning models comprising interconnected nodes, or neurons, organized in a layered structure [28]. ANNs are trained on large amounts of data and learn to recognize patterns and relationships within that data, allowing them to make predictions or classifications on new data [29].

In their review, Arshad et al. [30] concluded that the most common types of ANN algorithms in the HAR literature are convolutional neural networks (CNNs) and long short-term memory recurrent networks (LSTMs). CNNs are a subclass of ANNs that have convolutional layers with neurons that conduct convolution operations on discrete regions of the input map, extracting features that contain information about local patterns [29]. On the other hand, LSTMs are a type of recurrent neural network with layers that comprise cells that can retain data in an internal memory over time [31]. Apart from these networks, Luptáková et al. [32] adapted a Transformer, a model normally used in natural language processing and vision tasks, so that it could be trained with inertial signals from a smartphone.

As for vision-based HAR, two main approaches are taken into consideration: RGB-based and skeleton-based [17,33,34]. In RGB-based HAR, data from RGB cameras are processed by computer vision algorithms such as convolutional neural networks to recognize patterns and classify activities by learning spatial and temporal features from video sequences, often by incorporating multi-frame optical flow to capture motion and enhance recognition performance [35]. However, this approach is susceptible to variations in lighting, clothing, and other factors, which makes extracting meaningful information about body position and movement challenging, especially in the presence of camera motion or occlusion. Recent research, such as that of Wang et al. [36], addresses the limitation of camera motion by improving the performance of dense trajectories through the correction of their estimation, enabling a more efficient video representation for action recognition. On the other hand, HPE or skeleton-based HAR involves using cameras to capture skeletal data, representing the human body as interconnected joints, which are then tracked and recognized using depth-based computer vision techniques to estimate 3D joint positions from a single depth image [37]. Skeleton-based HAR is commonly preferred over RGB-based HAR since it provides more precise information about body position and movement, making activity identification easier, as 3D skeletal data are less affected by variations in appearance, lighting, and background noise [38]. Additionally, skeleton data have lower dimensionality, requiring less processing power and memory, making it suitable for real-time applications [39]. While occlusion is one of the most significant factors compromising the performance of vision-based HAR approaches, skeleton-based methods are more robust to this challenge. This topic has been the subject of study in the recent literature [40,41,42].

Therefore, handling image data presents several challenges due to variability in capture conditions, a common issue across all fields involving image processing and classification. This challenge is evident not only in HAR but also in areas such as inverse synthetic aperture radar (ISAR) target classification, where significant challenges include image deformation and few-shot classification problems [43]. Furthermore, recent advancements in semi-supervised learning, particularly the Evidential Tri-Branch Consistency learning framework (ETC-Net) for medical image segmentation, demonstrate significant potential for effectively leveraging limited labeled data. The ETC-Net showcases methodologies that could similarly enhance vision-based HAR systems, especially in scenarios where annotated data are scarce [44].

A common approach in HPE involves using 2D inference as a preliminary step to 3D estimation [45]. HPE algorithms can analyze either single-person or multi-person images, and two approaches are commonly used: top-down and bottom-up [46]. In the top-down approach, individuals in the image are first identified using detection algorithms and then the pose is predicted separately for each person. In the bottom-up approach, joint keypoints are first extracted and then grouped into individuals using clustering techniques. Top-down algorithms are generally more accurate but use more computational resources as they require two detectors, while bottom-up algorithms are faster but less accurate.

For 2D estimation, one of the most popular trends is the use of encoder–decoders as they facilitate the extraction of relevant abstract information to achieve the desired output. On the other hand, regression methods and heat-map-based methods may be distinguished [47]. The latter aims at predicting a keypoint heat map where each pixel indicates the probability that the keypoint is at that position. When generating the training data, a Gaussian function on the true data is used to compute these probabilities. Because of these characteristics, heat maps provide spatial information, which is relevant and facilitates the training of convolutional neural networks.

As for 3D estimation, the followed approach involves extracting the 3D pose from the common 2D pose with Fully Connected Networks [48], Temporal Convolutional Networks [49,50], Graph Neural Networks [51], or Transformer networks [52,53]. In this work, four architectures were implemented as the basis for 3D-HPE inference [50,53,54,55].

As previously mentioned, multimodal HAR combines data from multiple sources, such as cameras and sensors, to recognize and classify human activities more accurately. This type of HAR leverages the strengths of different modalities to overcome the limitations of individual approaches. Due to the dimensionality of the data, it is not trivial to combine the information obtained by various modality sensors, and therefore, the data collected for a multimodal HAR system can be fused at various phases. The most popular methods for fusing data from distinct modality sensors are data-level, feature-level, and decision-level fusion [19]. Data-level fusion combines raw data from various sensors. Feature-level fusion or early fusion [56] takes place after features are extracted from the raw data. Finally, decision-level fusion or late fusion happens when combining the decision of several classifiers, each of them working with a single modality. This type of fusion enables utilizing each modality’s strengths, which can produce superior recognition outcomes. However, late fusion takes more time and requires a complex learning process, which could result in a possible loss of inter-modality connection.

### 2.2. Related Frameworks

Across the state of the art, multiple frameworks used for either HAR or HPE may be found. As for HPE, MMPose [57] is an open-source pose estimation framework for easy training and deployment of human pose models. It is modular, supports different types of input data, includes multiple algorithms, and offers pretrained models and visualization tools. However, MMPose has potential disadvantages such as requiring technical expertise, particularly for beginners, and being complex and time consuming to configure due to its wide range of customization options. OpenPose [57] is another popular open-source library for real-time human pose estimation that uses deep learning and can detect and track multiple people in a single frame. It can estimate 2D and 3D poses, has multiple applications, and is known for its accuracy and speed.

Current state-of-the-art studies focusing on HAR cover diverse aspects. Some propose multimodal methods that fuse visual information with sensors, while others concentrate on the exclusive use and treatment of sensors such as IMUs, accelerometers, and gyroscopes. Despite the wealth of research suggesting various methodologies and data processing techniques for HAR, a significant proportion of these studies fail to make their solutions available as open source through a framework or library, which would offer a valuable resource to the scientific community.

Xaviar et al. [58] propose a multimodal sensor data fusion model for HAR that is robust against data quality issues. Their strength lies in offering an alternative approach that outperforms the state of the art, allowing for more optimal treatment in the presence of consecutive missing data and noise. Additionally, they have a repository containing the implementation of their study. However, unlike the library we propose, they do not focus on images or videos and do not implement approaches for pose estimation. Other authors, such as Ouyang et al. [59], focus on contrastive learning for HAR, providing a new system for contrastive fusion learning with small data in multimodal HAR applications. The authors offer their code freely. Mollyn et al. [60] propose a multimodal deep learning model that combines sensor and audio information. Their results demonstrate a competitive performance with an accuracy of 92.2% in 26 daily activities in four indoor environments. Although they provide a generic open-source approach, the recognized activities are not related to walking patterns, such as falls, which are the central focus of our proposal, where the incorporation of pose estimation algorithms is crucial.

Other approaches, like that of Noori et al. [61], focus on methods where the input consists of images. They propose an LSTM-RNN approach for HAR using the open-source library OpenPose for extracting anatomical keypoints. Although their proposed method shows a promising performance, with the best result achieving an overall accuracy of 92.4% on a publicly accessible dataset, surpassing conventional approaches like Support Vector Machines, Decision Trees, and Random Forests, we did not find their proposal in an open-source repository. Also using OpenPose, authors Duhme et al. [62] present an approach for multimodal action recognition, combining anatomical keypoints from OpenPose with IMU-based information and RGB data. They use other algorithms such as graph convolutional networks. While their study also shows comparable results, it relies solely on OpenPose for HPE.

More recently, Islam et al. [63] propose a multilevel feature fusion technique for HAR using a CNN to process visual data and ConvLSTM to handle information from multiple sensor sources. They experimented and validated their approach on the open-source UP-FALL multimodal dataset, which is the central focus of the evaluation in this article. Although the technique is promising, it faces certain challenges, such as the need for substantial computational resources. Additionally, the model is designed to require multimodal data, whereas single-mode data may be common in the real world.

In light of the limitations observed in current state-of-the-art studies, we propose the BodyFlow library as a comprehensive solution. This open-source benchmark is provided specifically for the scientific community to utilize in HAR research and development. BodyFlow integrates both the application of HPE algorithms and the exclusive consideration of sensor data, as well as their multimodal combination. In this manner, the library provides a multimodal and modular design that also caters to the specific needs of the user.

In comparison to established libraries, such as OpenPose and MMPose, BodyFlow distinguishes itself through its unique strength in modular design. This distinctive characteristic empowers users to selectively combine different networks for both HPE and HAR. Notably, BodyFlow offers a high degree of flexibility, allowing users to experiment with diverse configurations and architectures by choosing from various input data combinations. This adaptability is particularly advantageous, promising an improved performance and suitability across a spectrum of scenarios. Unlike some libraries with more rigid structures, BodyFlow’s modular approach shines in its customization potential, making it a versatile and promising solution for users seeking flexibility and enhanced performance in HPE and HAR.

Furthermore, BodyFlow facilitates rapid experimentation for researchers and developers working on HPE and HAR models. In this context, existing benchmarks, such as the one presented by Andriluka et al. [64], provide datasets for testing HPE and tracking algorithms. Additionally, there are benchmarks that assess datasets, models, and training strategies for solving HPE and HAR problems [65,66]. However, it is important to note that none of these approaches offer a benchmark toolbox comparable to BodyFlow, which allows researchers to create variations of their algorithms and thoroughly test them.

## 3. BodyFlow

BodyFlow is an open-source library that leverages cutting-edge deep learning techniques, including custom algorithms, to accurately estimate human poses in 2D and 3D and detect the activity performed.

**Figure 1:**
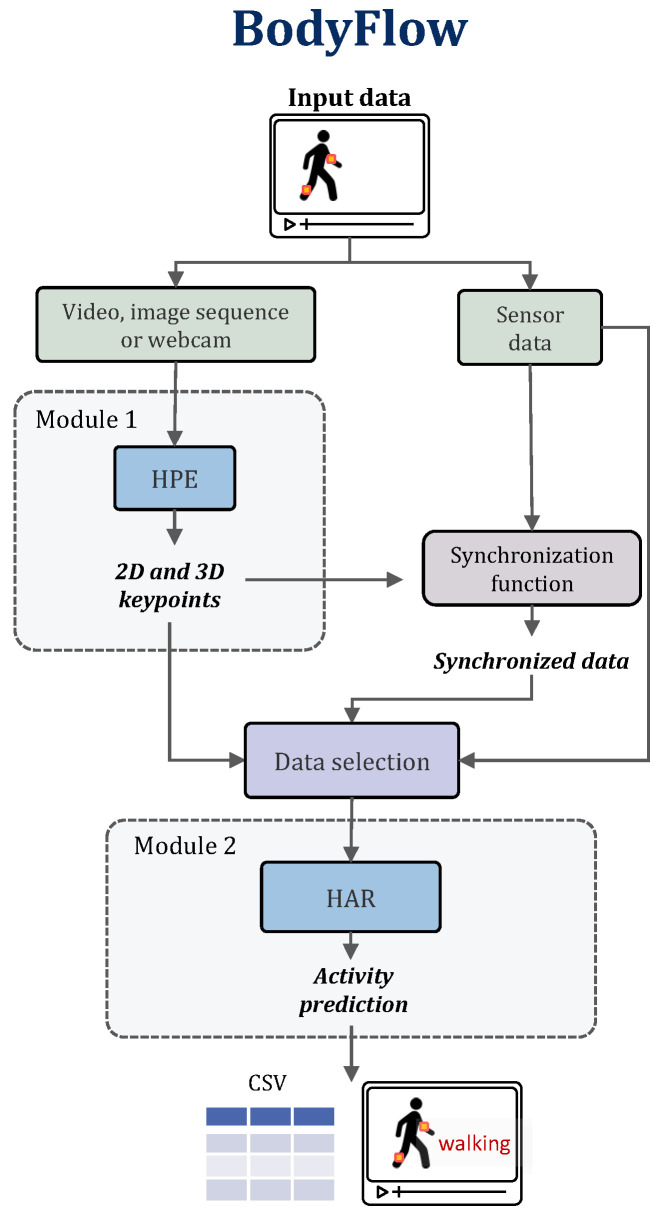
BodyFlow library framework.

The proposed library, a multimodal framework, comprises two main modules: human pose estimation (HPE) and human activity recognition (HAR), as illustrated in Figure 1. It is capable of managing visual and sensor data and their combinations. The visual data might be a video file, a sequence of images, or the input from a webcam. If only visual data are available, Module 1 infers the 2D and 3D keypoints and forwards the information to the HAR module, which predicts the activity. If only sensor data are available, these data are fed directly into the HAR module. When vision and sensor data are available, the first module outputs the keypoints, and a custom function synchronizes the keypoints with the sensor data. It is worth mentioning that the synchronization function must be customized for each dataset. BodyFlow has been tested with the publicly available multimodal UP-FALL dataset, which gathers data from 11 activities.

### 3.1. Module 1: Human Pose Estimation

**Figure 2:**
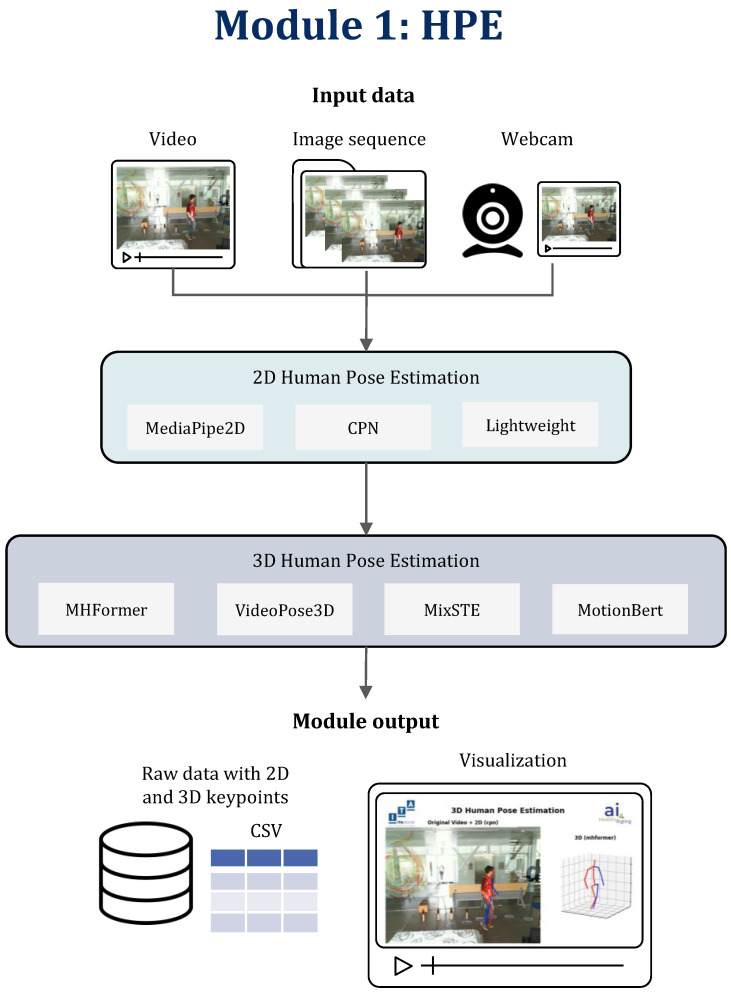
Human pose estimation module pipeline.

The HPE module is designed to process a variety of inputs, including video/webcam feeds or sets of images, as shown in Figure 2, and provides outputs in both 2D and 3D formats, depending on the user’s requirements. The top-down approach was selected primarily for its flexibility in seamlessly integrating the 2D pose detector and the subsequent 3D pose detector. This is conducted through a process called 2D lifting, where 2D keypoints (coordinates from the image) are projected into 3D space by estimating the missing depth dimension. Rather than relying on explicit visual cues like shadows or perspective, the system infers the 3D pose by analyzing the geometric relationships between 2D keypoints and correlating them with learned 3D body structures [50,53,54,55]. This conversion allows for more detailed spatial analysis, forming a critical link between 2D pose detection and 3D pose estimation, enabling the system to extract the three-dimensional body structure from simple 2D inputs efficiently. The use of keypoints in this process allows the system to focus on specific features of the human body (such as joints or body segments) without having to process the entire image or video in a two-dimensional way from the start. This not only reduces the computational load but also improves the pose detection accuracy as the system can concentrate on areas of interest. This strategic choice provides a spectrum of options for fine-tuning, allowing for the customization of a 3D pose predictor based on specific constraints, such as real-time processing, memory optimization, precision, and other factors. Furthermore, top-down methodologies commonly achieve promising results by leveraging state-of-the-art person detection techniques and concentrating on single-person human pose estimation, underscoring the efficacy of the chosen approach [46]. This decision aligns with our long-term objective of applying BodyFlow in medical research, where the highest level of accuracy is paramount. However, we also support Multi-Person Pose Estimation by detecting and tracking all individuals across frames and then performing single pose estimation on each detected person. While this feature is available as an extra, our analyses are conducted on single-person estimations to limit the scope of the work.

Users have the flexibility to select between different 2D and 3D algorithms for processing the input. In its initial stage, the module offers a selection of three distinct 2D detectors. Each of these detectors is capable of converting RGB monocular images into a comprehensive set of 2D body joints. These keypoints accurately represent the pixel coordinates of various body parts, such as the nose, hands, and hips. Subsequently, these 2D keypoints are elevated to a 3D representation by the module’s 3D detectors.

Three methods are included in BodyFlow for estimating 2D poses: the Cascade Pyramid Network (CPN) [67], lightweight [68], and MediaPipe Pose [69]. The framework’s 3D-HPE algorithms are VideoPose [50], MHFormer [70], Mixed Spatio-Temporal Encoder architecture (MixSTE) [53], and MotionBert [55]. For completeness, we also included ExPose [71] and MediaPipe Pose [69] for 3D-HPE. However, these two use an end-to-end approach rather than a top-down approach and are not included in any analyses.

First, the winners of the MSCOCO keypoints 2017 challenge [72] presented a novel network called the Cascade Pyramid Network (CPN) [67], composed of two smaller networks: GlobalNet and RefineNet [73]. Then, in [68], an architecture for HPE in a multi-person environment for peripheral devices is presented. The work is based on OpenPose [74] but with an optimized architecture, which obtains a higher accuracy due to modifications carried out on the same architecture. Lastly, MediaPipe Pose [69], based on BlazePose [75], is a lightweight and simple convolutional neural network, which infers 33 body keypoints for a single person. The network is based on the use of heat maps and linear regression to obtain the coordinates of each keypoint. This algorithm locates keypoints in both 2D and 3D.

As for 3D-HPE, a fully convolutional architecture based on temporal convolutions, which take as input a consecutive series of 2D poses called VideoPose, was presented in [50]. Among the advantages of said algorithm is that by using a wide input, subtle transitions between frames are generated with less susceptibility to noise, as well as the use of unlabeled data during training.

In 2019, a Transformer-based architecture called MHFormer [70], capable of learning spatio-temporal relationships from multiple pose hypotheses by performing three stages, was presented. The first stage is to generate a set of initial hypotheses, followed by modeling these hypotheses and merging multiple hypotheses into a convergent representation and partitioning them into divergent hypotheses. Then, it generates inter-hypothesis communication and aggregates the multi-hypothesis features to synthesize the final 3D pose.

Furthermore, the Mixed Spatio-Temporal Encoder architecture (MixSTE) [53] consists of a temporal Transformer that models the temporal motion of each keypoint and a spatial Transformer to learn the spatial relationships between keypoints.

The last 3D detector is MotionBert [55]: a motion encoder based on encoder–decoder training with various data sources is proposed that is able to recover 3D data from corrupted skeletons. This architecture, called the Dual-stream Spatio-Temporal Transformer, is able to become a practically universal motion analyzer that is able to analyze relationships between the keypoints of each skeleton, despite noisy conditions.

EXpressive POse and Shape rEgression (ExPose) estimates the 3D body pose, hand articulation, and facial expression from a single RGB image. Unlike previous methods that rely on optimization and 2D keypoints, ExPose directly regresses the body, face, and hands in SMPL-X format, addressing high dimensionality and the lack of expressive training data. It uses body-driven attention for higher-resolution crops of the face and hands, leveraging part-specific knowledge from existing datasets. ExPose offers accurate, expressive 3D human estimation with a reduced computational cost.

To achieve multi-person estimation, it is crucial to consistently identify the same individuals across all frames in a video. We included three tracking algorithms to facilitate this process, allowing users to select the most appropriate one for their specific requirements. The available algorithms are SORT [76], Deep SORT [77], and Bytrack [78].

The reported results for the top-down previously mentioned 2D to 3D lifting algorithms on the Human 3.6 M dataset [79] test set are presented in Table 1. The results are shown considering Protocol 1, the mean per joint position error (MPJPE), and Protocol 2, rigid alignment in post-processing (P-MPJPE).

BodyFlow’s modular design allows for flexibility in selecting and combining the networks of the pose estimation pipeline. Furthermore, it allows the user to add new models by adding the model class and the trained weights. This modularity makes it easy to experiment with different combinations of components to achieve the best performance for a given task. Results on the processing time of the different 2D and 3D model combinations are presented in Table 2. Even though the fastest combination is the lightweight and VideoPose3D models, the difference in inference is barely 10%.

### 3.2. Synchronization Function

**Figure 3:**
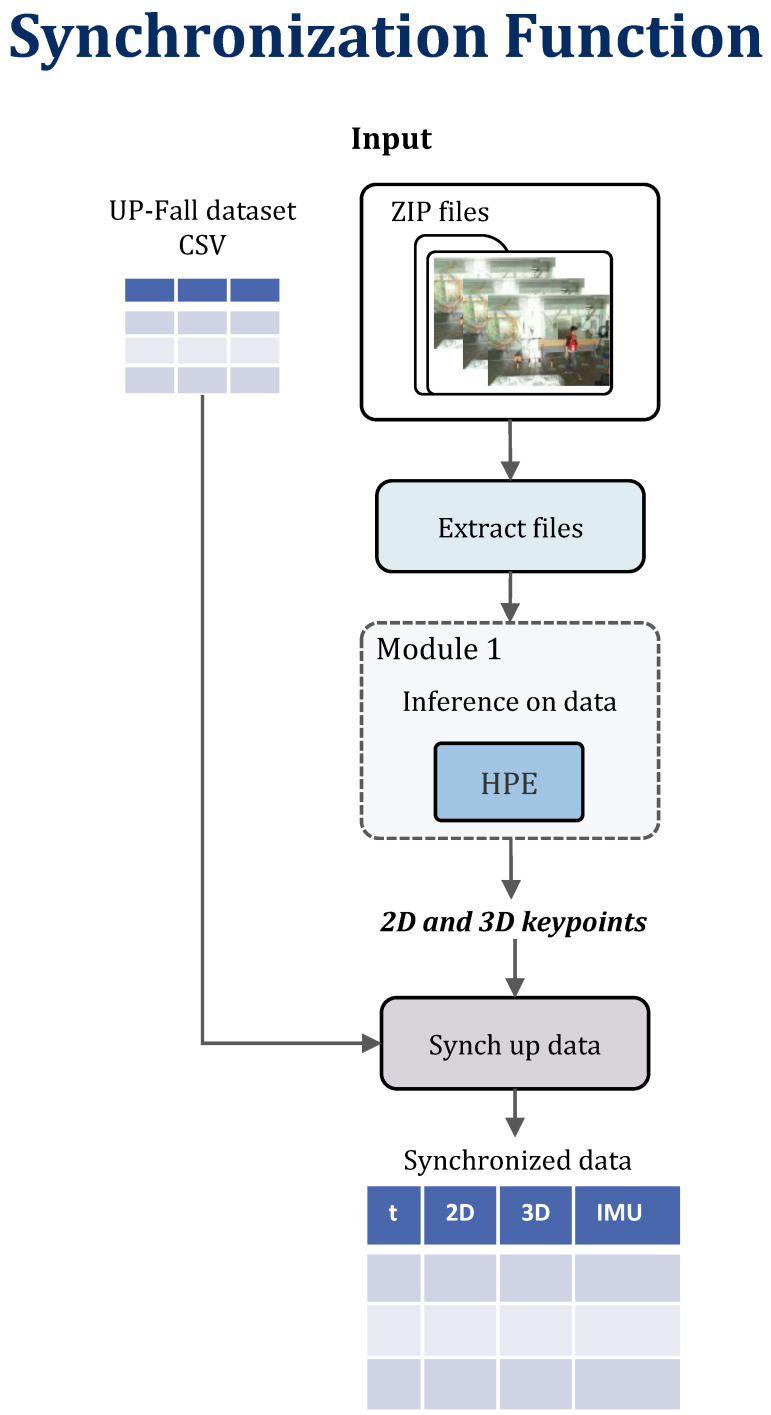
BodyFlow synchronization function.

**Figure 4:**
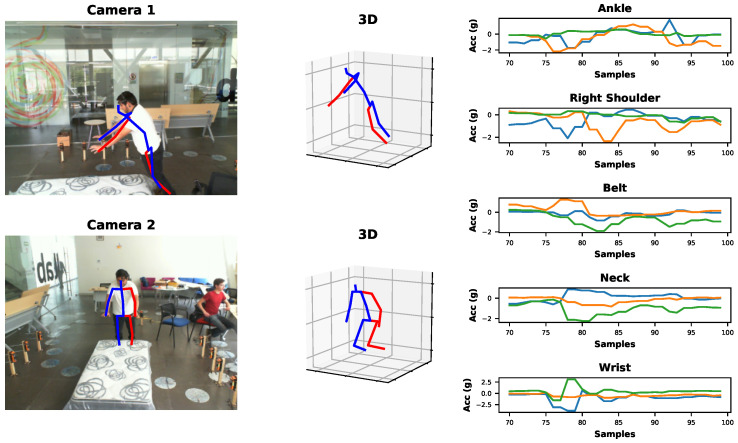
Synchronized data example of the activity falling in the UP-FALL dataset. On the (**left**) side, both camera views with 2D poses are shown. In the (**middle**), the 3D visualization is shown, and on the (**right**) side, the acceleration plots from sensor data are displayed, which are synchronized with the video.

The BodyFlow library incorporates a synchronization function responsible for performing data-level fusion of the input data, see Figure 3. In this process, Module 1 extracts the pose from a sequence of frames and subsequently combines this pose with the raw information obtained from the inertial sensors by using the timestamps of the frames. After the successful fusion, the synchronized data are saved into a single *.csv* file before being forwarded to the activity classifier of the HAR module. Figure 4 shows a visual example of the image data synchronized with inertial data from five sensors that the subject wore.

This merged file simplifies the training process in the HAR module as it allows for flexibility in selecting features. For example, the activity classifier can be trained with only sensor data or with a combination of sensor data and pose information, enabling more precise feature selection based on the desired model configuration.

Additionally, new methods for synchronizing custom data have been explored. All IMUs are synchronized with each other, so the primary challenge is synchronizing the IMUs with the camera. To achieve this, the video was synchronized with IMU data by hitting two metal bars, one of which had an IMU attached. The impact generated a distinct peak in the IMU data and a corresponding peak in the audio recorded by the camera, allowing for the accurate alignment of the data streams.

### 3.3. Module 2: Human Activity Recognition

**Figure 5:**
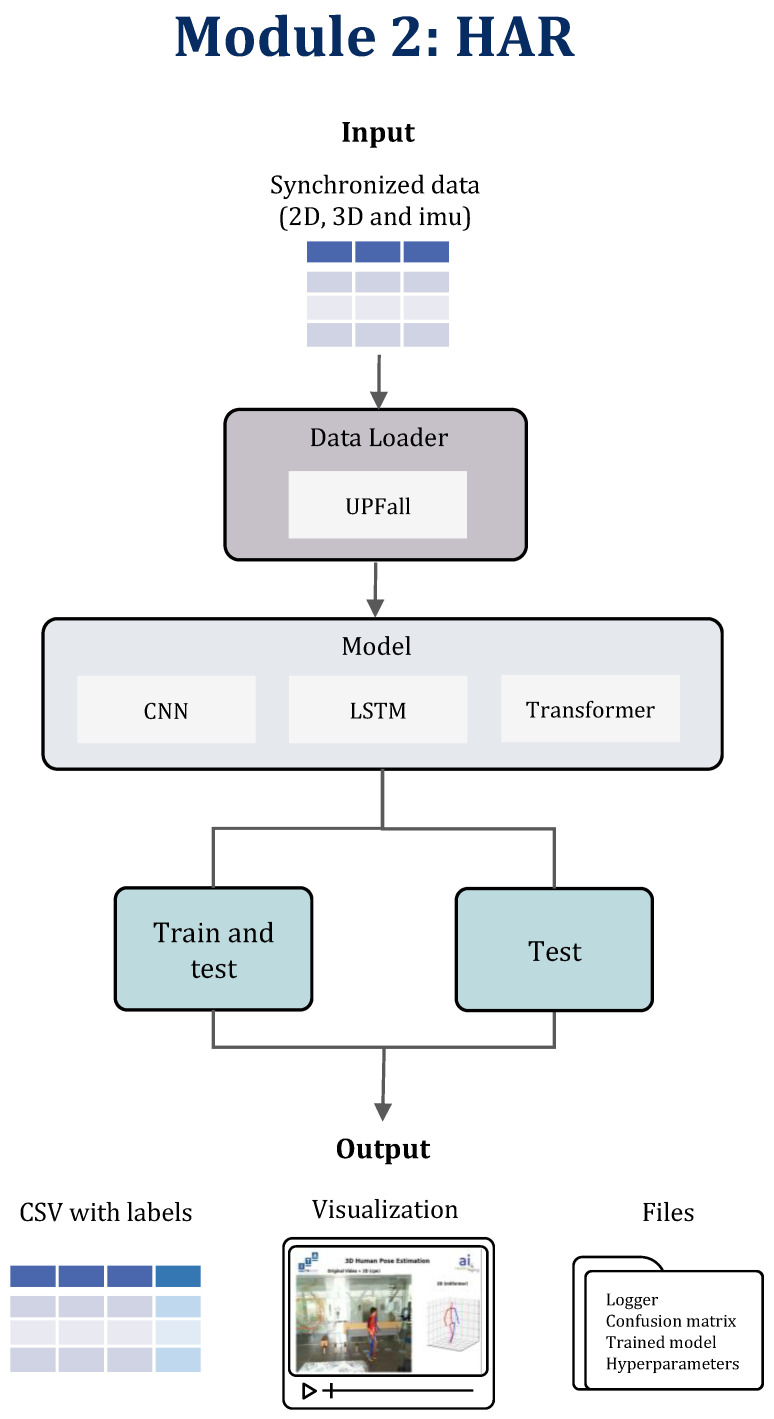
Human activity recognition pipeline.

The second module eases the training and evaluation of different neural network architectures. In this manner, the user may use input data, which contain 2D, 3D, or sensor data or a combination of any to train and/or evaluate using the UP-FALL dataset (Figure 5). The pretrained models are included in the library, but it is also possible to add custom data loaders and models to satisfy specific needs for any other task. Moreover, it is possible to evaluate using vision data only, making use of one of the pretrained models.

The user can select from three multiclassifier pretrained models of the library, all of which were implemented in PyTorch. These model architectures are a CNN (adapted from [80]), an LSTM, and a Transformer; the approximate size of each are presented in Table 3. It is worth mentioning that each of these models varies in size depending on the input size. Each model was trained by splitting the synchronized data into train, validation, and test sets, making use of a sliding window technique. The hyperparameters employed during training, derived from hyperparameter optimization, include the following settings: a batch size of 64, 100 training epochs, and the Adam optimizer. Additionally, distinct learning rates were assigned for each HAR model: 1 × 10^−3^ for the LSTM and CNN, and 1 × 10^−5^ for the Transformer. An early stopping method was implemented based on the minimum loss value of the validation set.

Furthermore, the library allows the user to choose from different combinations of input data, thus allowing for the search for the best combination of inputs, including (1) a 2D pose, (2) a 3D pose, (3) data from all inertial sensors, (4) data from one inertial sensor, and (5) data from all inertial sensors plus 2D and 3D poses.

A sliding window is a technique used in signal processing and machine learning for analyzing sequential data by breaking them down into shorter segments or windows of equal length. Thus, another variable that determines the size of the input is the length of the window or window size. The library allows one to select three window sizes: 21, 41, and 81. Since the UP-FALL dataset was recorded at 18 frames per second, these window sizes correspond, respectively, to 1.1, 2.2, and 4.5 s, respectively.

The BodyFlow HAR module implements two open-source libraries to facilitate the training and management of models: Pytorch Lightning [81] and MLflow [82]. PyTorch Lightning is a popular open-source framework that is designed to make it easier to train machine learning models with PyTorch. It provides a high-level interface for PyTorch that simplifies the process of training and evaluating deep learning models. On the other hand, MLflow is an open-source platform for managing the end-to-end machine learning life-cycle. MLflow, which also supports integration with Pytorch, helps simplify and accelerate the machine learning development process by providing a unified platform for managing experiments, models, and deployments.

The BodyFlow repository, located at https://github.com/ITAINNOVA/BodyFlow (accessed on 18 October 2024), provides access to the code and further information on its usage.

## 4. Results

As previously mentioned, the proposed library was evaluated with the publicly available UP-FALL dataset [18], which is a multimodal dataset that includes 11 different activities performed by 17 healthy young individuals, with each participant conducting three trials for each activity. In particular, the library was evaluated with five inertial sensors and the frames from both frontal and lateral cameras as input data. Furthermore, for this evaluation, trials 1 and 2 were used for training, while trial 3 was used for testing.

Table 4 presents the test results of our experiments on the UP-FALL dataset, examining various combinations of HAR models and input types. A comparison was also made between our results and those of three recent state-of-the-art studies, namely [18,80,83], which employed the same dataset to derive their findings. The evaluation metrics considered in this study include the macro F1-score, macro precision, macro recall, and accuracy. These metrics provide a comprehensive overview as they average the results for each class equally, regardless of their frequency or size in the dataset.

As mentioned above, the library allows for the usage of different combinations of features in order to study and analyze which type of sensor is more suitable to recognize activities. Specifically, the column *Features* of Table 4 shows which inputs were used for the HAR model: *3D*: the 3D pose from module 1, *2mD*: the 2D pose from module 1, *imus*: all five inertial sensors, *ankle*: only the inertial sensor placed on the ankle, and *all*: a fusion of the 2D pose, 3D pose, and all five inertial sensors. In particular, these results were obtained using the 2D CPN pose algorithm, the 3D MHFormer pose algorithm, and a window size of 21 frames.

The results indicate that, on the whole, integrating multimodal data from images and IMUs leads to an improved classification performance in comparison to using unimodal data. In general, relying solely on the inertial sensor information from the ankle yields the poorest performance. The standout model is the Transformer, achieving a macro F1-score of 0.815, although the LSTM closely follows.

In terms of comparing our results with state-of-the-art studies, when considering accuracy as the evaluation metric, we observed that our best-performing model outperforms the results presented by Suarez et al. [80] and Espinosa et al. [83]. However, it is noteworthy that the highest overall accuracy is achieved by Martínez-Villasenor [18]. Additionally, it should be taken into account that accuracy can be a misleading metric when classes are imbalanced, since a model can achieve high accuracy simply by predicting the most frequent class. The UP-FALL dataset is an example of an unbalanced dataset, where there are many more walking events than events of a specific fall, for example. The F1-score is a more robust metric, especially when addressing class imbalances, as it effectively balances precision and recall. When the F1-score is used to evaluate our model, a significant outperformance is observed in comparison to the studies conducted by Espinosa et al. [83] and Martínez-Villasenor et al. [18]. The highest F1-score is attained by Suarez et al. [80], albeit our results are notably close to theirs.

**Figure 6:**
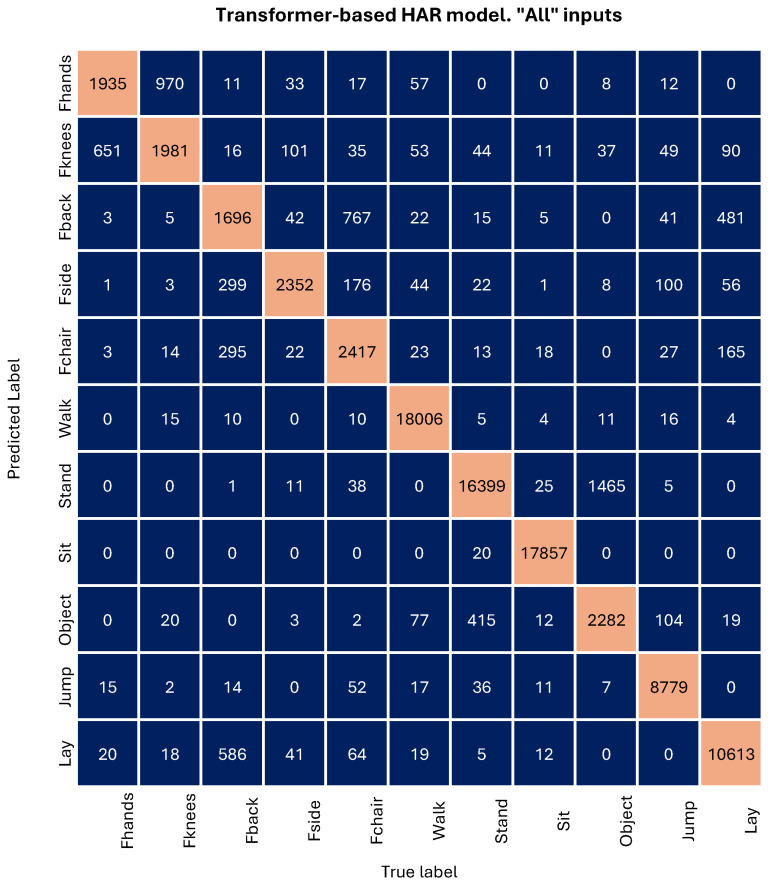
(1) FHands = falling forward using hands, (2) Fknees = falling forward using knees, (3) Fback = falling backward, (4) Fside = falling sideward, (5) Fchair = falling sitting in empty chair, (6) Walk = walking, (7) Stand = standing, (8) Sit = sitting, (9) Object = picking up an object, (10) Jump = jumping, (11) Lay = laying.

Figure 6 presents the confusion matrix generated by the library for the model that achieved the highest performance on the test set; specifically, the Transformer model trained with data from both IMUs and cameras. These matrices serve as summaries of the classification algorithm’s performance, enabling users to make more effective result comparisons. In the present context, each column within the matrix corresponds to the actual (true) labels of activities, while each row represents the predicted labels generated by the HAR model. The intersection of a row and column in the matrix denotes the count of instances where the actual label matches the predicted label.

It is evident from the matrix of Figure 6 that the five types of falls are the worst classified compared to the other activities. This discrepancy arises from an unbalanced dataset, where there is five times more data on non-fall activities than on any of the falls.

Upon closer examination of the falling activities, a discernible pattern emerges based on the nature of each activity. Falling forward using hands and falling forward using knees exhibit striking similarities. The subject begins by standing in front of a mattress for a few seconds and then falls, remaining in a prone position for additional seconds. The fall, inherently brief, poses a challenge for the model to distinguish the pattern of standing, falling forward (with or without the use of knees), and lying down.

Similarly, falling backward and falling sitting in an empty chair undergo a comparable pattern. The person starts standing with their back to the mattress, falls (also in a similar manner), and lies down face up on the mattress. In this case, there is observed confusion not only between these two activities but also with the activity of lying down (being supine).

Finally, it is worth noting the confusion between the activities “Object” and “Stand”. The activity “Object” refers to picking up an object and consists of three phases: standing, picking up a small object from the floor, and standing again. The fact that the standing sub-activity is predominant leads to this confusion.

In summary, the results show that the BodyFlow library enabled the development of HAR models with various architectures and types of data, achieving the detection of 11 activities with a performance comparable to or exceeding that of other state-of-the-art models. It is also observed that fall detection is more challenging due to its inherent complexity and possibly its fleeting nature, which can make it resemble a combination of activities such as walking, stumbling, and lying down or sitting.

## 5. Discussion and Conclusions

HAR brings notable benefits in the field of health, such as assisting the elderly through the real-time monitoring of their activities, promoting autonomy, preventing risks, promoting the early detection of degenerative diseases, and laying the groundwork for personalized and early interventions through the analysis of activity patterns.

The integration of diverse sources of information, including the fusion of visual data from cameras with inertial sensors or wearable device information, holds the potential to offer more detailed and accurate representations of human activities. Nonetheless, effectively managing and integrating data from various sources is challenging. The successful integration of these technologies requires advanced image processing approaches, deep learning techniques, and data fusion algorithms, contributing to the development of more robust and accurate multimodal HAR systems in real-world environments. Moreover, HPE can play a significant role in human activity recognition but also encounters difficulties, including variability in poses, multiple camera views and angles, changes in lighting, and the presence of multiple people in the scene, issues that are being addressed in the specialized literature.

This paper presents BodyFlow, a library designed to smoothly integrate HPE and HAR methods in open-source code. During its development, various state-of-the-art methods and techniques were examined, carefully selecting those most suitable that are now available in the library.

BodyFlow exhibits several notable strengths. One is the ability to address multimodality, allowing for the combination of visual data processed by HPE algorithms with inertial sensor information through a custom synchronization function. Another key advantage of BodyFlow lies in its modular design, giving users the ability to select and combine various networks for human pose estimation and human activity recognition. It also allows users to choose from various input data combinations. This flexibility enables experimentation with different configurations and architectures, potentially leading to an improved performance and adaptability to different scenarios.

The integration of PyTorch Lightning and MLflow into BodyFlow provides additional benefits by simplifying experimentation, training, and model management. Ultimately, BodyFlow was built with the goal of enabling researchers and developers to quickly experiment with different HPE and HAR models, as well as with various input sources such as inertial sensors and multi-camera videos. This experimentation capability with various component combinations provides modularity and specialization for specific tasks.

BodyFlow was assessed using the UP-FALL dataset, an open dataset widely used for HAR tasks [18,80,83]. Experiments were conducted with various combinations of input data and HAR models. Among these, the Transformer HAR model, trained on data from both IMU sensors and cameras, yielded the most favorable macro metrics, emphasizing the value of integrating multimodal information. Our macro metrics, including the F1-score, precision, and recall, achieved values of 0.815, 0.816, and 0.819, respectively, surpassing the results reported in [18,83]. However, our performance slightly trailed behind Suarez et al. [80], which obtained an F1-score of 0.836. Their study utilized a CNN HAR model incorporating pose data from camera 2 (the frontal view), estimated with MediaPipe, along with the skeleton features distance and velocity.

Martínez-Villasenor et al.’s study [18] achieved the highest accuracy (0.951), though this was based on a CNN model that did not incorporate pose estimation. While their approach achieved notable accuracy, particularly in common activities such as standing and laying, it showed weaker results in fall-related activities, where our multimodal approach demonstrated a superior performance. For example, in the activity of falling sitting in an empty chair, our model achieved an accuracy of 0.76, outperforming the 0.26 reported by Martínez-Villasenor et al.

Espinosa et al. [83] proposed a fall detection system based on multiple cameras and convolutional neural networks (CNNs), achieving an F1-score of 0.729 and a precision of 0.742. However, our multimodal approach, which integrates both visual and inertial data, significantly outperforms their results, with our Transformer model achieving an F1-score of 0.815 and a precision of 0.816. This highlights the advantage of multimodal data fusion over using visual data alone.

It is worth noting that [18] did not provide comprehensive details regarding dataset partitioning, mentioning the use of 140,451 samples for testing and 70,145 samples for training. In the study conducted, a similar approach to that of [80] was followed, using trials 1 and 2 for training and trial 3 for testing. While our metrics may not claim the top position, they still demonstrate a satisfactory performance, aligning with the state of the art.

To use BodyFlow with custom data, the visual and IMU information should be synchronized first. The UP-FALL dataset is already synchronized with timestamps, so this step was not needed. There exist different alternatives for achieving this synchronization. One approach involves utilizing a common clock, either through software synchronization or with the aid of a hardware device, a technique commonly employed in cinematography for synchronizing different cameras. Alternatively, a physical cue, such as creating a stamp with the foot or clap, presents a second approach. This action registers as a peak in the corresponding IMU, while the sound is concurrently recorded by the camera. The synchronization process involves aligning the IMU peak with the sound peak [84].

On the other hand, BodyFlow is currently in its first version and has some limitations that will be addressed in future releases. Future work will include analyzing the performance of other algorithms implemented for HPE, which are expected to improve on the results presented. It will also include the ability to train the HPE module with custom-made datasets, allowing for greater adaptability to specific domains. This could be particularly important in healthcare, where the ability to train the HPE with domain-specific data can lead to more accurate and personalized health monitoring and diagnostic tools. For example, in physical rehabilitation, a custom-trained HPE could analyze patient movements more precisely, helping healthcare providers track recovery progress and adjust treatment plans based on the individual’s specific needs. Additionally, future work will also focus on analyzing the performance of other algorithms implemented for HPE, which are expected to further improve the results presented, enhancing the system’s overall accuracy and efficiency. Regarding synchronization, future work will aim to improve the synchronization function to support a broader range of data sources beyond the UP-FALL dataset. This enhancement will streamline the integration of libraries that rely on different formats, sampling rates, or modalities.

Furthermore, despite the fact that the user has the option to infer on visual data only, the addition of a generic synchronization function may benefit the use of different data sources. Additionally, our ongoing work on a feature engineering module—highlighted through the comparison with Suarez et al.’s study [80]—underlines the crucial role of feature engineering, with features such as the distance, velocity, and angles of key body parts having the potential to significantly enhance our classification capabilities. The exploration of algorithm combinations, such as CNN-LSTM networks, is also considered in the planning of future work.

In addition to the aforementioned initiatives, focused on improving functionality, capacity, and library evaluation, an additional goal is to leverage BodyFlow capabilities to address real health problems in elderly people, such as predicting degenerative diseases, gait analysis, and other related conditions. Similarly, as part of our future work, we plan to conduct extensive testing of the BodyFlow library on a broader range of datasets, extending beyond the UP-FALL dataset. This approach aims to assess the generalizability and robustness of our library across diverse scenarios and activity contexts. By incorporating additional datasets, we can further validate the performance of BodyFlow and ensure its effectiveness in various real-world applications.

Despite the potential for future enhancements and additional features, we want to emphasize that BodyFlow is already a stable and functional solution, capable of achieving satisfactory results in its current state. Our intention is to provide a robust and effective tool that, in the meantime, serves as a solid foundation for future research and development in these fields.

Our library is open source, allowing for free access and continuous improvement in its functionalities. It offers a modular, flexible, and multimodal system, unlike other tools. Although there are studies on HAR in the health sector, real applications are lacking, and many authors do not share their code for various reasons, such as privacy concerns. Aznar et al. [1] present their methodology for detecting user behaviors and preventing health issues, but they do not have an open-source library, and their approach is limited to sensor data. In contrast, our library allows for the integration of visual information through HPE techniques, enriching the analytical capabilities. Dao et al. [85] and Zhang et al. [86] developed fall detection methods using pose models and shared their code on GitHub. However, their approaches focus solely on image use. In this regard, our work stands out for its multimodal approach, which allows for the combination of sensor data and images. Other studies, such as those by Cheng et al. [12], focus on detecting neurodegenerative diseases like Parkinson’s using data from smartphone sensors. These unimodal approaches can be enhanced by the tools in our library, which includes pose detection, thus providing additional analysis that may be crucial for diagnosing and monitoring these conditions.

In the future, we plan to expand our library with additional modules to detect diseases such as sarcopenia or cognitive decline like Parkinson’s disease, where HPE and HAR techniques can be particularly beneficial for this purpose. Maachi et al. [87] propose an intelligent system, proving their code on Github, for analyzing gait data from Parkinson’s patients that relies solely on sensor data and does not incorporate pose estimation. Our library could be integrated to perform HPE, thereby enhancing the system.

In conclusion, BodyFlow is a promising tool in the field of HPE and HAR. Its strengths in modularity, flexibility, and multimodality are noteworthy for researchers interested in both fields. To the best of our knowledge, there is currently no open-source library that integrates HPE and HAR with these advantages. The functionalities offered by the library allow for the detection of both human pose and the activities performed, information that could help identify potential health risks and facilitate their prevention.

## Figures and Tables

**Table 1:** 3D-HPE networks considering Human 3.6 M dataset.

Algorithm	Protocol 1	Protocol 2	No. Parameters
VideoPose3D (2019)	46.8 mm	36.5 mm	16.95 M
MHFormer (2022)	43.1 mm	34.5 mm	25.15 M
MixSTE (2022)	40.9 mm	32.6 mm	33.78 M
MotionBert (2022)	39.1 mm	33.2 mm	16 M

**Table 2:** HPE module inference time.

3D/2D	MediaPipe2D	CPN	Lightweight
VideoPose3D	0.92	0.94	0.90
MHFormer	1	0.95	0.95
MixSTE	0.96	0.97	0.95
MotionBert	0.99	1	0.97

**Table 3:** Architecture comparison of the models.

Algorithm	Number of Layers	No. Parameters	Size (MB)
CNN	16	2.3 M	10
LSTM	8	1.6 M	8
Transformer	56	135 M	540

**Table 4:** UP-FALL dataset results. Macro metrics.

HAR Model	Features	F1	Accuracy	Precision	Recall
LSTM	All	0.811	0.904	0.813	0.813
LSTM	3D	0.792	0.898	0.804	0.784
LSTM	2D	0.476	0.682	0.649	0.473
LSTM	IMUs	0.783	0.887	0.807	0.766
LSTM	Ankle	0.572	0.744	0.576	0.572
CNN	All	0.749	0.887	0.751	0.752
CNN	3D	0.772	0.885	0.772	0.776
CNN	2D	0.763	0.865	0.766	0.765
CNN	IMUs	0.698	0.856	0.737	0.688
CNN	Ankle	0.603	0.788	0.623	0.595
Transformer	All	0.815	0.913	0.816	0.819
Transformer	3D	0.766	0.871	0.758	0.778
Transformer	2D	0.769	0.877	0.767	0.778
Transformer	IMUs	0.778	0.887	0.793	0.767
Transformer	Ankle	0.611	0.777	0.613	0.612
Espinosa et al. [83]		0.729	0.822	0.742	0.716
Martinez-Villasenor et al. [18]		0.712	**0.951**	0.718	0.713
Suarez et al. [80]		**0.836**	0.893	**0.848**	**0.834**

Bold highlights the highest discrimination capacity considering the F1-score metric, accuracy, precision, or recall.

## Data Availability

The public dataset used for this study can be found at https://sites.google.com/up.edu.mx/har-up/ [18] (accessed on 22 August 2024).

## Abbreviations

The following abbreviations are used in this manuscript:

ANN	Artificial Neural Network
CNN	Convolutional neural network
CPN	Cascade Pyramid Network
HAR	Human activity recognition
HPE	Human pose estimation
IMU	Inertial measurement unit
LSTM	Long short-term memory
